# Work Stress and Altered Biomarkers: A Synthesis of Findings Based on the Effort–Reward Imbalance Model

**DOI:** 10.3390/ijerph14111373

**Published:** 2017-11-10

**Authors:** Johannes Siegrist, Jian Li

**Affiliations:** 1 Life Science Centre, University of Düsseldorf, Merowingerplatz 1a, 40225 Düsseldorf, Germany; 2 Institute of Occupational, Social and Environmental Medicine, Centre for Health and Society, Faculty of Medicine, University of Düsseldorf, Universitätsstrasse 1, 40225 Düsseldorf, Germany; jian.li@uni-duesseldorf.de

**Keywords:** effort-reward imbalance, over-commitment, biomarkers, work stress, narrative review

## Abstract

While epidemiological studies provide statistical evidence on associations of exposures such as stressful work with elevated risks of stress-related disorders (e.g., coronary heart disease or depression), additional information on biological pathways and biomarkers underlying these associations is required. In this contribution, we summarize the current state of the art on research findings linking stressful work, in terms of an established theoretical model—effort-reward imbalance—with a broad range of biomarkers. Based on structured electronic literature search and recent available systematic reviews, our synthesis of findings indicates that associations of work stress with heart rate variability, altered blood lipids, and risk of metabolic syndrome are rather consistent and robust. Significant relationships with blood pressure, heart rate, altered immune function and inflammation, cortisol release, and haemostatic biomarkers were also observed, but due to conflicting findings additional data will be needed to reach a firm conclusion. This narrative review of empirical evidence supports the argument that the biomarkers under study can act as mediators of epidemiologically established associations of work stress, as measured by effort–reward imbalance, with incident stress-related disorders.

## 1. Introduction

Linking exposure data on adverse working conditions with the risk of incident disease in prospective cohort studies is considered to be a major source of evidence in occupational health research. Importantly, these studies aim at meeting the criteria of establishing a causal association between adverse work and ill health, as defined by Bradford Hill [1]. One such criterion—the demonstration of biological pathways between exposure and disease development—is particularly relevant, as it adds biological plausibility to the statistical associations [2]. Over the past few decades, emphasis on occupational exposures with impact on workers’ health has shifted from physical and chemical hazards to adverse psychosocial work environments [3]. At least three factors contributed to this shift.

First, with significant changes in the nature of work and employment in a post-industrialized, globalized labour market, distinct psycho-mental and socio-emotional stressors became more widespread and more visible, and their impact on health and wellbeing was increasingly recognized. Second, significant progress was achieved in identifying crucial aspects of a health-adverse psychosocial work environment through theoretical models and their psychometrically validated measures. Three such models received special attention: “job demand-control” [4], “effort-reward imbalance” [5], and “organizational injustice” [6]. These complementary models differ in terms of their focus within the complexities of work environments. The “demand-control” model is concerned with job task characteristics defined by high psychological demands in combination with a low degree of personal control and skill discretion. “Effort-reward imbalance” focuses on violations of the principle of social reciprocity inherent in the work contract, where high efforts spent are not reciprocated by adequate rewards (money, esteem, promotion, job security). Moreover, in addition to the two extrinsic (situational) components “effort” and “reward”, the model maintains that an intrinsic (personal) component—a motivational pattern of high job involvement (“over-commitment”)—contributes to adverse health outcomes. The third model, “organizational injustice”, is devoted to the study of procedural, interactional, and distributive inequities among people working in organizations.

The initiation of pioneering occupational cohort studies linking adverse working conditions to elevated long-term risks of morbidity and mortality was a third factor of this shift. The British Whitehall II study [7] and the French GAZEL study [8] are influential examples of this shift, especially so as they implemented measures of stressful work. Meanwhile, a substantial evidence base has been established on associations of stressful work with elevated risks of incident stress-related disorders in a variety of occupations and national workforces (for coronary heart disease see [9,10]; for depression see [11,12,13]; for a general review see [14].

However, knowledge on psychobiological processes as the pathways “through which psychosocial factors stimulate biological systems via central nervous system activation of autonomic, neuroendocrine, immune, and inflammatory responses” is still limited [15] (p. 103). In the long run, these pathways increase the pathology of stress-related disorders, such as cardiovascular diseases, metabolic disorders, and affective disorders, through their sustained altered activity levels that trigger allostatic load in distinct organ systems [2,16,17,18]. It is therefore a major task to identify those biomarkers that can be altered by exposure to stressful work, and to review research findings that document respective associations.

In this contribution, we provide an updated synthesis of available results on relationships between work stress (as measured by effort–reward imbalance) and a range of biomarkers whose alterations in terms of dysregulated physiological responses contribute to allostatic load and pathophysiological developments. For the following reasons, the model of effort-reward imbalance (ERI) was chosen as a focus of analysis. First, this model fits with a general notion of a psychosocial work environment with relevance to health that has been defined as “the interaction between a person’s cognitions, emotions, and behaviours, and the material and social work context” [19] (p. 100). Unlike the other concepts of stressful work introduced above, this model (as mentioned) includes an intrinsic (personal) component (“over-commitment”), thus enabling an analysis of the interaction of situational and personal components. More specifically, research findings on associations with health indicators are either restricted to the two extrinsic components or their combination (effort-reward imbalance (ERI), defined as ratio or as interaction term), or they additionally include the intrinsic component (in terms of direct effect or moderation effect) [20]. Second, with its emphasis on work pressure, job insecurity, limited promotion prospects, and poor pay, the ERI model may specifically cover some relevant trends of occupational life in the context of economic globalization and rapid technological change [10]. Third, in recent years, research on ERI and altered biomarkers was rapidly growing, thus justifying an attempt to synthesize and critically discuss current knowledge.

## 2. Materials and Methods

This contribution provides an overview of research findings published in original scientific contributions, review papers, or book chapters. Although we applied an established literature search strategy (see below), it does not represent a systematic review and meta-analysis. Rather, we draw major information from five recent systematic reviews [21,22,23,24,25], from a recent book chapter [26], and from an additional electronic search in the databases PubMed and PsycINFO. This literature search included the time period from 1 January 2011 until 31 August 2017 (by Jian Li). In line with the previous systematic reviews and book chapter, the following list of search terms was applied: (“ERI” or “effort reward imbalance” or “reward” or “overcommitment” or “over-commitment”) and (“cardiac electrophysiology” or “vagal tone” or “heart rate” or “heart rate variability” or “HRV” or “catecholamines” or “adrenaline” or “epinephrine” or “noradrenaline” or “norepinephrine” or “cortisol” or “ACTH” or “adrenocorticotropic hormone” or “CAR” or “cortisol awakening response” or “DHEA” or “dehydroepiandrosterone” or “CD4” or “CD8” or “t-helper cell” or “IFN” or “interferon” or “IL” or “interleukin” or “NKC” or “natural killer cell” or “immunoglobulin” or “TNF” or “tumor necrosis factor” or “CRP” or “C-reactive protein” or “BP” or “blood pressure” or “hypertension” or “ambulatory BP” or “systolic BP” or “diastolic BP” or “fibrinogen” or “dyslipidemia” or “lipids” or “cholesterol” or “HbA1c” or “glycated haemoglobin” or “metabolic syndrome” or “allostatic load”).

In our literature search, only peer-reviewed English-language articles with original data included. We identified 1606 papers, and after the exclusion of titles already repeatedly identified across databases PubMed and PsycINFO, 383 abstracts and full papers were screened, and 35 papers were finally added to our analysis. Due to the large number of single studies and the heterogeneity of reporting results, this presentation does not apply a unified quality assessment of the studies, nor does it list the detailed findings in systematized table overviews. Rather, we provide a descriptive synthesis of main findings for each single biomarker and, in case of metabolic syndrome, for combined measures.

## 3. Results

This section is organized according to main classes of biomarkers that mirror neural, (neuro-) endocrine, cardiovascular, metabolic, inflammatory, and immune responses to stressful stimuli. While short-term activation of these responses is considered an adaptive reaction of the organism, recurrent chronic activation may alter their function and trigger dysregulated physiologic developments that ultimately result in structural changes and overt disease of distinct organ systems [2]. Although relatively subtle and small, stress responses that are registered in laboratory experiments, in real life ambulatory monitoring devices, or as part of a biomedical screening in epidemiological studies become manifest as deviations from normal levels or patterns (increase or decrease; altered secretion pattern, delayed recovery rate, etc.), and it is their reduced adaptability associated with cumulative change that matters in the long run [15]. In the frame of the “allostatic load” concept, it was proposed to distinguish between primary (e.g., catecholamine secretion) and secondary mediators (e.g., cumulative visceral fat) as well as tertiary outcomes (e.g., metabolic syndrome; type 2 diabetes) within individual stress trajectories [27]. However, in this paper we focus on biomarkers of early stages. Thus, while changes in blood pressure are considered, we do not include studies linking work stress with the prevalence or incidence of hypertension or of clinical consequences such as left ventricular hypertrophy. Equally, increased inflammation is included, but not carotid atherosclerosis. 

With our focus on early stages of a stress trajectory, evidence on the following four classes of biomarkers is presented: 1. biomarkers related to activation of the sympatho-adrenal medullary axis; 2. biomarkers related to activation of the hypothalamic-pituitary-adrenocortical axis; 3. biomarkers related to the immune response and inflammation; and 4. metabolic and haemostatic biomarkers.

### 3.1. Biomarkers of the Sympatho-Adrenal Axis and Cardiovascular Biomarkers

Biological responses resulting from stress-induced activation of structures in the prefrontal cortex and limbic system are mainly organized through the sympatho-adrenal medullary (SAM) axis and the hypothalamic-pituitary-adrenocortical (HPA) axis [28]. SAM-related activation includes the release of peripheral adrenaline and noradrenaline hormones as well as differential sympathetic and parasympathetic (vagal) arousal, as manifest in altered heart rate (HR), heart rate variability (HRV) and blood pressure (BP).

HR was assessed in relation to ERI in two studies using ambulatory monitoring and in two laboratory stress experiments. Vrijkotte et al. [29] observed increased HR during work, as registered over two workdays, in employees of a computer company who displayed high vs. low ERI (*p* = 0.01). When ERI was measured by event momentary assessment in a group of 100 nurses, a significant association with HR was observed [30]. In a within-group experimental design with a reward vs. standard condition in 60 women in Australia, significantly smaller increases in HR following a challenging task in the reward vs. standard condition were observed [31]. It is of interest to know that one of the earliest experimental studies on ERI and HR was conducted in a group of middle-aged industrial middle managers with high, medium, and low chronic work stress. HR increase was monitored during a modified colour-word interference task. There was a significant trend where higher work stress was associated with lower maximal HR responsiveness [32]. This finding was interpreted as a functional adaptation to excessive stimulation through downregulation of adrenoceptors [33]. Taken together, while associations of ERI with HR were observed in three out of four studies, it seems important to specify stages within the trajectory of stress exposure, suggesting that HR is increased following challenge in an early stage, but reduced in a late stage of chronic exposure, due to adaptive change to excessive stimulation.

In several studies, ambulatory blood pressure (ABP) was assessed during one or several working days [29,34,35], or even repeatedly in the frame of a prospective epidemiological study over up to seven years [36,37,38,39,40] in association with work stress measures. One of these studies performed in a group of 74 female and 26 male employees in Italy found no association of ERI with elevated ABP over two working days [35], whereas in the Vrijkotte et al. study [29], an imbalance between effort and reward was associated with an average increase in systolic blood pressure (SBP) of 4 mmHg, and this increase was not restricted to work time, but persisted during leisure time and non-work days. Among British civil servants, SBP over a working day was significantly higher among those scoring high on over-commitment, and this effect was particularly strong in the subgroup with the lowest occupational position [34].

The Canadian prospective study included up to 3395 white collar male and female workers, and several reports are available from this landmark study, using different time frames and sub-samples. In short, after 3 years, women scoring high on ERI had a significant increase in systolic ABP or, among older women, a significantly increased incidence of hypertension, whereas no significant effect was observed among men [36]. This trend among women was confirmed over a 5-year observation period [38]. However, when cross-sectional associations of ERI and over-commitment with ABP were analysed subsequently several times, some associations were also detected among men [37]. Importantly, the double exposure of ERI and family obligations among women resulted in a significant rise in ABP after 5 years [40]. Moreover, it is instructive to know that in this prospective study, ERI was associated with significantly increased risks of untreated hypertension [39] and masked hypertension [41].

Two more studies deserve attention: one dealing with change in BP during pregnancy where an association with ERI and over-commitment was observed [42], and a laboratory study using an acute stressor where altered BP during exposure was not significantly associated with altered BP [32]. As mentioned, the inconsistent findings from a number of cross-sectional studies on ERI and prevalence of high BP or hypertension were not included in this analysis. Overall, the reported results on ABP support the notion that failed reciprocity at work elicits BP increases with potentially adverse long-term effects.

Very few studies have explored an association of ERI with catecholamine secretion in acute psychosocial stress tasks. In the study on industrial middle-managers, a negative association between high work stress and change in adrenaline (but not noradrenaline) from baseline to maximal challenge was observed [32]. In line with the notion of compromised sympathetic responsiveness, another study of 58 healthy men found that noradrenaline secretion in response to a standardized social stress test was significantly reduced among those scoring high on over-commitment [43]. Another biomarker of enhanced SAM activity—salivary alpha amylase (sAA)—was analysed in the Australian within-group experiment mentioned, and this response was significantly reduced in the “reward” vs. standard condition [31]. However, given the paucity of findings, no firm conclusion can be drawn.

HRV is a further, more widely examined, biomarker of enhanced autonomic nervous system (ANS) activation. This indicator reflects the balance between sympathetic and vagal influences on heart rate [44]. Low HRV mirrors a sympathetic predominance and a decreased vagal tone, thus reducing the restorative capacity of ANS [45]. Decreased cardiac vagal tone is considered an early sign of functional impairment of the cardiovascular system, and several studies demonstrate an association with increased prospective cardiovascular morbidity and mortality [46,47,48]. Complementary measures of HRV have been proposed, and RMSSD (the root mean square of successive differences of the inter-beat-interval series) is one of the frequently applied robust indicators of parasympathetic cardiac control [49]. In this brief review, we do not describe the different indicators used in the studies, nor can we make direct comparisons of findings, given the variety of measures and the differences of registration time of HRV across studies. We identified ten studies reporting data on the ERI model and HRV. Eight studies used a cross-sectional design and two studies an experimental design. In nine studies, a significantly negative association of ERI components with HRV was found, but in some reports with gender differences (women only: [50,51]; men only: [52]), or with age differences (middle-aged subjects only: [53]). Extrinsic components were more often associated with reduced HRV than over-commitment (e.g., [54]), but one study reported a significant effect of the intrinsic component, but no association with extrinsic components [55]. Another study reported significant negative effects for either component [56]. One study was of borderline significance (*p* = 0.059) [29]. In the Mannheim study on industrial workers, ERI was indirectly associated with glycemic measures mediated by HRV [57]. Remarkably, the findings of the two experimental studies confirmed this negative association [31,58], thus supporting the hypothesis that chronic experience of stressful work has a dysregulatory effect on ANS balance.

In conclusion, findings derived from ambulatory monitoring studies of BP and HRV as well as from a few laboratory experiments demonstrate a rather consistent association of components of the ERI model with alterations of these two biomarkers, whereas the results on HR and on hormonal markers of the SAM axis are currently not conclusive.

### 3.2. Biomarkers of the Hypothalamic-Pituitary-Adrenocortical (HPA) Axis

As a central control system within the organism, the HPA axis regulates several important physiological functions, such as energy mobilization, glucose production, release of free fatty acids, water balance, and functioning of the immune system [27]. Cortisol plays a primary role in this regulation, and its pattern of diurnal secretion is modified, among others, by chronic psychosocial stress. Substantial research was conducted on associations of work stress with cortisol secretion, with a recent emphasis on ERI. It is challenging to summarize research findings, as several indicators of cortisol were proposed (cortisol awakening response (CAR), awaking cortisol concentration (ACC), cortisol diurnal pattern (CDP), afternoon or evening cortisol). Moreover, samples are taken from plasma, urine, hair, or, most commonly, saliva. As substantial gender differences were reported in associations of work stress with cortisol, any brief account of findings seems difficult. In an excellent recent systematic review and meta-analysis, Eddy et al. [22] summarized the results of 14 studies testing associations of ERI with cortisol, as well as 10 including data on over-commitment [51,59,60,61,62,63,64,65,66,67,68,69,70,71,72,73,74,75]. In either gender (but more pronounced among men), an increase in CAR or elevated ACC was associated with ERI in a majority of studies, but often bypassing statistical significance. Yet, in the meta-analysis, a significant summary effect was observed. Over-commitment was less consistently related to these two markers, but more so to elevated cortisol concentration in the afternoon and evening [22]. Most studies were based on saliva cortisol, but two recent studies report analysed data with hair cortisol [74,75], where one report could not confirm the association [75]. This meta-analysis included single studies with additional hormones related to HPA axis (dehydroepiandrosterone, DHEA; adrenocorticotropic hormone, ACTH), but it is premature to extend this scope.

In our literature search, we identified a few additional studies that partially support the results of this meta-analysis. Among participants scoring high on over-commitment, Steptoe et al. [34] found elevated diurnal cortisol release, and Marchand et al. [76] reported an elevated CAR. In another study, high ACC was observed among men with high ERI [77]. A recent study from Australia found a significantly positive association of effort-reward ratio with CAR [78]. One report found a relatively weak negative association of ERI with salivary cortisol at different times during the day or night [79], and one investigation reported a relation of the amount of change in job insecurity over two measurement waves—a subcomponent of reward—with hair cortisol at second measurement [80]. Yet, two investigations reported either a negative relationship [81] or lack of association [82].

In view of a negative feedback action of cortisol in regulating the HPA axis that suppresses the secretion of ACTH from the anterior pituitary gland, an additional potential impact of stress-related cortisol release on HPA dysregulation was investigated. In a study of teachers, Bellingrath et al. [64] observed that work stress in terms of low reward was associated with stronger cortisol suppression after low-dose dexamethasone application, thus pointing to altered HPA axis negative feedback sensitivity. A similar effect was observed among study participants with high over-commitment [63]. In contrast, high over-commitment was related to high elevated cortisol (but not ACTH) secretion in a combined dexamethasone/CRH test in a further study [67]. However, this finding could not be replicated ([73]; for discussion see [26]). Despite these inconsistencies, there is reason to assume that chronic exposure to psychosocial stress may contribute to a state of hypo-responsiveness of the HPA axis (see also [32]).

In sum, the current state of evidence by and large supports the conclusion of Eddy et al. [22] that components of the ERI model of stressful work are associated with altered responsiveness of the HPA axis, as measured by different markers of cortisol release. However, due to considerable inconsistency in single studies, further investigation on this association is required.

### 3.3. Biomarkers of Immune Function and Inflammation

Several reviews assessed an association of different types of chronic psychosocial stress with immune function [83,84]. A broad range of biomarkers of cellular and humoral immunity has been investigated in these studies, in particular counts or toxicity of natural killer (NK) cells, counts of CD4+ T cells, CD4:CD8 ratio, several cytotoxic T lymphocyte (CTL) subsets, and serum immunoglobulin G (IgG) or secretory immunoglobulin A [85,86]. A general finding indicates that chronic stress goes along with reduced immuno-competence and accelerated immune-senescence. As inflammatory cytokines are involved in the regulation of the human immune response, associations of chronic stress with pro- or anti-inflammatory cytokines have been studied as well [26]. Important biomarkers include interleukin (IL)-6, IL-10, IL-2, tumor necrosis factor (TNF)-a, and the acute phase protein C-reactive protein (CRP). In accordance with the above hypothesis, chronic stress is expected to result in increased pro-inflammatory and reduced anti-inflammatory activity [69].

In 2012, Nakata published a systematic review on current knowledge on the impact of chronic work stress on immune function, where different concepts were included (e.g., demand–control, organizational injustice, organizational downsizing, unemployment, economic recession, as well as effort-reward imbalance [86]). While the findings were generally in line with the hypotheses mentioned, more extensive research is currently available specifically with regard to ERI at work. Therefore, we summarize the results from two recent reviews [21,26], and we complement them by the most recent evidence. 

In a study of 347 Japanese men and women, a significant negative association of extrinsic components of the ERI model with counts and toxicity of NKC and a positive association with counts of B cells was observed, but only among men. The number of NKC was reduced by about 20 percent in the stressed group, compared to the group with low or no work-related stress. Associations with over-commitment were no longer significant in the fully adjusted model, and no associations were observed among women [87]. In a German study of 537 factory workers, several markers of immune-senescence were associated with components of the ERI model, most markedly low reward and low social support at work was an additional relevant predictor [85]. Secretory immunoglobulin A (sIgA), a marker of mucosal immunity, was clearly reduced in participants with high effort and low reward in two Australian studies, but only one study found an association with over-commitment [68,70]. Yet, in a group of law enforcement officers, a high level of IL-1, IL-6, and TNF-a, and a low level of IL-10 was not related to ERI [88].

Studies that apply acute mental stress tests are particularly instructive, as they allow for testing the responsiveness of immune and inflammatory markers. In an experiment with 55 men and women, Bellingrath et al. [89] assessed TNF-a and a series of interleukin markers before and after an acute laboratory stressor. Scoring high on ERI was related to an overall increase of pro-inflammatory activity and a decrease of anti-inflammatory IL-10 after stress. This finding was supported by a further experiment in 46 healthy schoolteachers, where high ERI was again associated with pro-inflammatory activity and where the capacity of dexamethasone to suppress IL-6 production was weakened before and after acute stress [90]. Mental stress testing was also performed in a British study of healthy middle-aged men. Participants with high effort-reward imbalance exhibited a significantly stronger increase in CRP under acute laboratory stress compared to those with low levels of work stress [91]. In two cross-sectional studies, one conducted among 204 Jordanian men [69] and one conducted in 731 working men and women in China [92], CRP was significantly associated with ERI, and in China additionally with over-commitment. More recently, baseline screening data from a large cohort of working men and women in France (*N* = 43,593) were analysed where high ERI was associated with higher white blood cell counts—a marker of increased low-grade systemic inflammation—both among men and women [93].

Taken together, there is supportive evidence that failed reciprocity at work in terms of high effort in combination with low reward is associated with altered functions of several immune and inflammatory markers, thereby eventually increasing the vulnerability to stress-related disorders. Effects are stronger among men and are more consistent with regard to the model’s extrinsic components. Aspects of low reward at work seem particularly important. Yet, given some conflicting findings and reduced comparability across studies, and given the cross-sectional design of several studies, additional investigations are required to reach firm conclusions.

### 3.4. Metabolic and Haemostatic Biomarkers

In a system-biological approach to the analysis of links between psychosocial stress and altered biomarkers, a web of causation involving distinct endocrine, immunologic, inflammatory, metabolic, and haemostatic factors is apparent. For instance, CRP and pro-inflammatory cytokines, activated by increased cortisol, affect insulin resistance and dyslipidemia. Resistance to insulin-stimulated glucose uptake is associated with an imbalance in blood coagulation and fibrinolysis. An elevated risk of metabolic syndrome seems to result from these interactions of metabolic and haemostatic dysregulation [69,94]. Therefore, it may be instructive to analyse associations of each one of the relevant metabolic and haemostatic biomarkers with indicators of stressful work, and to complement this analysis by introducing more comprehensive measures, such as indicators of metabolic syndrome.

Concerning haemostatic (and fibrinolytic) biomarkers, available reports assessed plasma fibrinogen, impaired fibrinolysis (increased type-1 plasminogen activator inhibitor (PAI-1); tissue-type plasminogen activator (tPA)), and D-dimer (indicating activation of the entire coagulation system) as main indicators. Inconsistent findings are available on relationships of ERI with fibrinogen. Two cross-sectional studies document significant associations [95,96], but other investigations failed to replicate this association [65,94,97]. However, in an impressive in-depth analysis of three-wave data from 124 middle-aged white collar workers in The Netherlands, Vrijkotte et al. [94] showed that the intrinsic component (over-commitment) was associated with an impaired fibrinolytic system, as manifested in decreased tPA and increased PAI-1, after adjusting for a broad range of confounders. Exhaustive coping at work seems to impair fibrinolytic activity within the organism. This conclusion is supported by the results of an investigation of 52 healthy teachers in Germany who were exposed to a standardized psychosocial stressor [66]. During recovery from stress, scoring high on over-commitment, but not scoring high on ERI, was related to an increase in the coagulation-enhancing plasma D-dimer and to a smaller decrease of fibrinogen. It is of interest to note that haemostatic factors seem to be more closely associated with the intrinsic than extrinsic component of the ERI model. This conclusion is further supported by the fact that one of the cross-sectional studies found strong associations with over-commitment among women, in addition to those reported on ERI [96].

Dyslipidemia is a major metabolic factor associated with an increased cardiovascular risk [98]. Since the classic study of Friedman et al. published in 1958 [99], atherogenic lipids have been associated with work-related stress, and the contribution of enhanced activation of SAM and HPA stress axes to the development of dyslipidemia has been convincingly demonstrated in many studies (e.g., [100]). Although there are some conflicting results (e.g., [101]) a majority of epidemiological studies support this association, either for the job strain model [102] or for the ERI model. In this latter case, the findings from seven studies are in line with this notion. In one of the earliest publications on components of the ERI model, the combination of economic downsizing and job insecurity among industrial blue-collar workers in Germany was associated with an adverse development of atherogenic lipids (low-density lipoprotein/high-density lipoprotein cholesterol (LDL/HDL) ratio) over 2 years [103]. In the Swedish WOLF study, effort and reward were related to elevated atherogenic lipids in men, while this association was observed with over-commitment among women [97]. Four smaller studies in China [104], Japan [60,105], and Germany [95], as well as a large recent French study [93] confirm the statistical association between ERI and an unfavourable lipid profile, with some noticeable gender variation.

As excessive catabolic activity involving the release of catecholamines and cortisol increases glucose production within liver cells, hyperglycemia is a probable consequence of HPA-mediated stress arousal [106]. Glycated haemoglobin (HbA1c) reflects serum-glucose concentrations over a couple of past weeks, and is a valid biomarker of diabetic risk; several investigations established a link of stressful work with elevated HbA1c [26,106]. To date, this association with work stress in terms of ERI is still weak, as results from only two studies are available. In a Chinese study, an association of ERI with HbA1c was restricted to female workers [107], and in a cross-sectional investigation of German industrial workers, ERI was linked to a measure of pre-diabetes, as defined by HbA1c, elevated glucose, and self-report data [108]. Baseline data from the French CONSTANCES study documented an association of ERI with elevated blood glucose among men, but the significance was lost after adjustment for relevant covariates [93].

A more reliable empirical basis is available if indicators of the metabolic syndrome are considered. An association of work stress in terms of job strain with metabolic syndrome was established several years ago [109]. However, evidence on a respective role of ERI was scarce until more recently, although a first attempt towards developing an index of this syndrome in association with the ERI model was undertaken by Vrijkotte et al. [94]. In this study, a risk score defined by nine parameters (glucose, insulin, three lipid measures, four fibrinolytic/haemostatic indicators) was significantly associated with a high level of over-commitment. The findings of five recent epidemiological reports from Germany [110], Italy [111,112], China [113], and South Korea [114] provide additional support of the hypothesis that work stress in terms of ERI is a risk factor of metabolic syndrome. Yet, only one additional study conducted in a sample of 204 Jordanian men that included data on cortisol and inflammation could demonstrate a plausible link between work stress, saliva cortisol, CRP, and risk of metabolic syndrome (MtS). In the group stratified according to high ER-ratio and upper tertile of high cortisol, the prevalence of MtS was 62.7 percent, compared to 20.8 percent in the non-stressed group, and it was 55.0 percent vs. 5.0 percent if stratified according to high ER-ratio and upper tertile of CRP [69].

Taken together, given a rather extensive basis of empirical evidence, there is considerable support of the hypothesis that effort–reward imbalance at work contributes to significant alterations of a range of metabolic and haemostatic biomarkers, and thereby may increase the risk of subsequent pathological developments.

### 3.5. Extending or Focusing the Range of Biomarkers?

This review has mainly dealt with single biomarkers, although the leading paradigm of research on stress and disease—allostatic load (AL)—maintains that a coordinated and sequential pattern of physiological responses is ultimately shaping the pathophysiological process [16,27]. Therefore, a comprehensive list of biomarkers representing this pattern of cumulative burden needs to be analysed in a simultaneous systemic approach. Although this argument is convincing, it nevertheless confronts researchers with several challenges. For instance, up to now, there is no consensus on a definite list of biomarkers to be included nor on the definition of thresholds required for the construction of a standardized, universally comparable AL index [115]. There is also no consensus on standardized measurement approaches (e.g., blood, urine, saliva samples; timing of data collection, etc.). Furthermore, it is questionable whether the same pattern of biomarkers predicts different types of stress-related disorders or whether disease-specific AL-constellations need to be investigated. Several studies using AL indices have not yet resolved this issue, and predictive evidence is limited. This also holds true for the few studies testing associations of ERI with AL [65,116].

Finally, with respect to future research development in this field, one may question whether a further extension of the range of biomarkers is the best approach, or whether it is more promising to focus scientific efforts on the search for those basic molecular biomarkers that initiate and promote the physiological and pathophysiological responses under consideration. For instance, it was proposed that oxidative stress plays a key role in this cascade of altered physiological processes, and that mitochondrial functioning deserves special attention [117,118]. While current evidence on a mediating role of oxidative stress in linking work stress with stress-related disorders such as coronary heart disease is still scarce and controversial [118,119], intensified interdisciplinary research along these lines is welcome.

## 4. Discussion

This report provides a synthesis of research findings on associations between single components or summary measures of the effort-reward imbalance (ERI) model of stressful work and a range of biomarkers with relevance to the development of stress-related chronic disorders. It is based on 67 papers published in peer-reviewed journals between 1988 and 2017 (31 August), covering publications in English language. The material was collected from a systematic electronic literature search, available systematic reviews and meta-analyses, and book chapters. In a majority of cases, significant associations of work stress in terms of the extrinsic and/or intrinsic components of the ERI model with the biomarkers under study were observed. Reduced heart rate variability (9 out of 10 studies), altered blood lipids (7 out of 9 studies), and increased markers of metabolic syndrome (6 out of 7 studies) were most consistently related to stressful work, although with gender variations. Increases in ambulatory blood pressure were associated in the landmark prospective Canadian study among women—in part in association with family obligations, although some smaller studies did not observe this pattern. Less consistency of findings or a small number of studies do not allow any conclusion with respect to links of ERI with heart rate, altered catecholamine secretion, and elevated fibrinogen. A remarkable amount of evidence concerns links of ERI with cortisol secretion as well as with markers of reduced immune competence and increased inflammation. In two meta-analyses, support of a significant overall effect of ERI on markers of the HPA axis and on markers of reduced immune competence was reported, and several additional studies support this conclusion, including some evidence on elevated inflammatory markers (CRP). However, given marked differences in study protocols, more research is needed on these latter associations.

This synthesis of empirical evidence supports the argument that these biomarkers can act as mediators of epidemiologically established associations of work stress exposure with incident stress-related disorders, such as coronary heart disease, type 2 diabetes, or depression. Thus, it is in line with an important criterion of causality in epidemiologic studies: the demonstration of biological pathways. The level of evidence of reported results is relatively high because many associations have been replicated in independent investigations, and consistency of several associations of work stress with biomarkers was observed in studies conducted in different countries and in varying occupational groups (references in Table 1). In summary, this new knowledge confirms and extends an available substantial body of research on distinct chronic psychosocial stressors and biomarkers, as documented in the case of social isolation, lack of social support, and loneliness [120] or in the case of a complimentary model of stressful work and job strain [106]. However, distinct from these concepts, the ERI model offers an explicit distinction between extrinsic and intrinsic components [20]. The findings of this review show that either component contributes independently to the explanation of altered biomarkers, and a few studies also show combined effects of these components.

Several unresolved issues call for further research. First, any generalization of our findings may be compromised due to publication bias. Although publication bias was addressed in the two meta-analytic reviews without finding strong evidence, and although a number of negative findings were published in this research, it is generally assumed that significant results have a higher chance of being submitted for publication than non-significant ones. A second unresolved issue concerns the temporal sequence of reported associations. Most findings are based on cross-sectional studies, laboratory experiments, or ambulatory monitoring. With a few noticeable exceptions, in particular the Canadian blood pressure study, exposure assessment was restricted to one or at least two measurement waves. The same holds true for data on altered biomarkers. It is therefore unclear whether this knowledge can be directly translated into a model of pathophysiological development linking exposure with disease onset. Third, the current state of the art tells little about the contribution of specific biomarkers to specific stress-related disorders. Are all of them involved in similar ways in the pathophysiology of these disorders? Is the broad concept of allostatic load the most promising approach towards tackling this challenge? Or do we need more basic research to unravel more fundamental molecular mechanisms and markers, as briefly discussed in the case of oxidative stress? Should research focus more intensely on the basic regulatory mechanisms in the brain rather than on peripheral markers of the organism? Clearly, these open questions deserve attention in future research.

This comprehensive updated synthesis of research on ERI and biomarkers suffers from several limitations in addition to its strengths. First, given the heterogeneity of study designs, the wide range of biomarkers, including their different assessment, and variation in statistical analyses of the data, we were not in a position to conduct a systematic review and meta-analysis according to established quality criteria. This next step of assessing cumulative evidence on the research question under study is considered a priority of future scientific enquiry. Second, by restricting the literature search to publications in English language, we may have bypassed some important contributions. However, most journals dealing with the topic under study publish their papers in the English language. In an additional effort, one of the authors (Johannes Siegrist) reviewed several books and journals of interest in German and French language, but did not find original scientific reports dealing with ERI and biomarkers. A further limitation of this review is related to an almost exclusive concentration to one type of stressful exposure—effort-reward imbalance at work, as experienced at the time of one or several assessments. With one exception exploring the simultaneous impact of work stress and family obligations on health [40], neither the cumulative load of co-occurring stressors nor a potentially disadvantaged life course preceding the experience of actual work stress have been addressed in this research, therefore eventually underestimating the contribution of failed reciprocity at work to the alteration of biomarkers. For instance, early life disadvantage was shown to be associated with a hazardous occupational trajectory and a high level of work-related stress [121], and accumulated deprivation over the life course, including low income and stressful work, contributes to an elevated mortality risk in early old age [122].

## 5. Conclusions

In this comprehensive synthesis of current evidence on associations of stressful work in terms of effort–reward imbalance with a range of altered biomarkers, we found preliminary support in favour of the assumption that altered biomarkers are involved in pathways leading from exposure to disadvantaged work to incident stress-related disorders. If further confirmed, these findings can instruct targeted preventive measures of identifying and diminishing stressful work.

## Figures and Tables

**Table 1:** Summary of effort–reward imbalance model and biomarkers.

Biomarkers	Extrinsic Component	Intrinsic Component	Number of Studies #
Sympatho-adrenal axis and cardiovascular system			
Heart rate	↑: Three [29,30,31] ↓: One [32]		Four [29,30,31,32]
Blood pressure	↑: Three [29,36,37] –: Two [32,35]	↑: One [34] –: One [29]	Six [29,32,34,35,36,37]
Heart rate variability	↓: Nine [29,31,50,51,52,53,54,56,58] –: One [55]	↓: Two [55,56]	Ten [29,31,50,51,52,53,54,55,56,58]
Hypothalamic–pituitary–adrenocortical axis			
Cortisol (majorly in saliva)	↑: Eight [51,61,69,74,76,77,78,80] ↓: Five [64,68,71,73,79] –: Eight [60,62,65,66,70,75,81,82]	↑: Three [34,63,76] –: Seven [59,60,64,66,67,70,72]	Twenty-six [34,51,59,60,61,62,63,64,65,66,67,68,69,70,71,72,73,74,75,76,77,78,79,80,81,82]
Immune function and inflammation			
Immune function	↓: Five [68,70,85,87,89] –: One [88]	↓: One [70] –: Two [68,87]	Six [68,70,85,87,88,89]
Inflammation	↑: Six [69,89,90,91,92,93]	↑: One [92] –: One [89]	Six [69,89,90,91,92,93]
Metabolic and haemostatic function			
Fibrinogen/impaired fibrinolysis	↑: Two [95,96] –: Four [65,66,94,97]	↑: Three [66,94,96]	Six [65,66,94,95,96,97]
Dyslipidemia	↑: Seven [60,93,95,97,103,104,105] –: Two [101,102]	↑: Two [97,104] –: One [60]	Nine [60,93,95,97,101,102,103,104,105]
Metabolic syndrome	↑: Six [69,110,111,112,113,114]	↑: One [94] –: One [112]	Seven [69,94,110,111,112,113,114]

↑: significant and positive association; ↓: significant and negative association; –: non-significant or null association. #: Some studies have investigated more than one association.
